# On Picturing a Candle: The Prehistory of Imagery Science

**DOI:** 10.3389/fpsyg.2016.00515

**Published:** 2016-04-19

**Authors:** Matthew MacKisack, Susan Aldworth, Fiona Macpherson, John Onians, Crawford Winlove, Adam Zeman

**Affiliations:** ^1^University of Exeter Medical SchoolExeter, UK; ^2^Artist-in-Residence, University of YorkYork, UK; ^3^University of GlasgowGlasgow, UK; ^4^University of East AngliaNorwich, UK

**Keywords:** visual imagery, fMRI, imagination, philosophy of mind, history of philosophy, history of psychology

## Abstract

The past 25 years have seen a rapid growth of knowledge about brain mechanisms involved in visual mental imagery. These advances have largely been made independently of the long history of philosophical – and even psychological – reckoning with imagery and its parent concept ‘imagination’. We suggest that the view from these empirical findings can be widened by an appreciation of imagination’s intellectual history, and we seek to show how that history both created the conditions for – and presents challenges to – the scientific endeavor. We focus on the neuroscientific literature’s most commonly used task – *imagining a concrete object –* and, after sketching what is known of the neurobiological mechanisms involved, we examine the same basic act of imagining from the perspective of several key positions in the history of philosophy and psychology. We present positions that, firstly, contextualize and inform the neuroscientific account, and secondly, pose conceptual and methodological challenges to the scientific analysis of imagery. We conclude by reflecting on the intellectual history of visualization in the light of contemporary science, and the extent to which such science may resolve long-standing theoretical debates.

## Introduction

Recent methodological developments have overcome longstanding obstacles to enable direct investigation of the neurobiology of mental imagery (Pearson et al., 2015). Technologies such as functional magnetic resonance imaging (fMRI) and positron emission tomography (PET) have fed the formation of a significant body of knowledge about visual mental imagery’s neural underpinnings. Researchers have shown, for example, that visualizing draws on many – but not all – of the same mechanisms as visual perception. Meanwhile, the nature and role of imagery has been a matter of keen philosophical interest since antiquity. By setting out this history, the following aims to inform and challenge the neuroscientific approach to visual mental imagery. It also aims to draw out the implications of the neuroscientific findings for researchers in other fields.

Even a cursory glance reveals how contested a capacity the ‘mind’s eye’ has been. We see, for example, that whereas Plato held the imagination in low regard, Aristotle claimed that the images produced by the imagination or *phantasia* are essential to thought: “The soul never thinks without a *phantasma*”. Aristotle’s (1984, 431a) views of the imagination and imagery pervaded thinking on the subject through the medieval period until Descartes (1985) in the 17th-century, who placed imaginings with sensations, on the side of the body – “a special way of thinking for material things” (Descartes, 1985, 6:37) – and so open to doubt. Kant, by contrast, re-iterated the centrality of the imagination to cognition, and introduced the link to *creativity* that would be extolled by the Romantic poets of the 19th-century. While for Kant the workings of the imagination were essentially mysterious – “arts concealed in the depths of the human soul” (Kant, 1998, p. 273) – Galton’s (1880) questionnaire *on visualizing and other allied activities* introduced the possibility that acts of imagining could be quantified and examined using modern scientific techniques: experimental psychology developed into a distinct academic discipline with introspection at its center. Mid-twentieth-century behaviorism threw out imagination and imagery as scientifically inadmissible; for its founder J. B. Watson, mental images were ultimately reducible to “motor habits in the larynx”. Cognitive psychology refocussed scientific attention on imagery with the development of experiments by Alan Paivio, Roger Shepard, Stephen Kosslyn, and others, which suggested aspects of thought that critically *depended* on mental phenomena such as imagery. Although in the 20th-century philosophers of both ‘analytic’ and ‘continental’ persuasion developed a host of models and theories about the imagination’s working, the *empirical* study of imagining took a decisive turn in the 1990s, with the development of neuroimaging technologies that make the brain’s processes visible.

Broadly, the resulting studies show that during visual imagery there is activation of a subset of the posterior (occipital and temporal) regions which are also engaged by visual perception – as shown by, for example, Kosslyn et al. (1993, 1997). Unlike perception, however, visual imagery coincides primarily with activation of more anterior (frontal and parietal) regions involved in executive function and attention (see Ishai et al., 2000), in keeping with its complex relationship with working memory. For many scientists, such as Behrmann (2000) and Kosslyn et al. (2001), these and other findings have provided definitive answers to questions that millennia of philosophical discussion about the imagination could only pose – around such issues as its relationship to perception and memory, how images are generated, even what images *are*.

**FIGURE 1:**
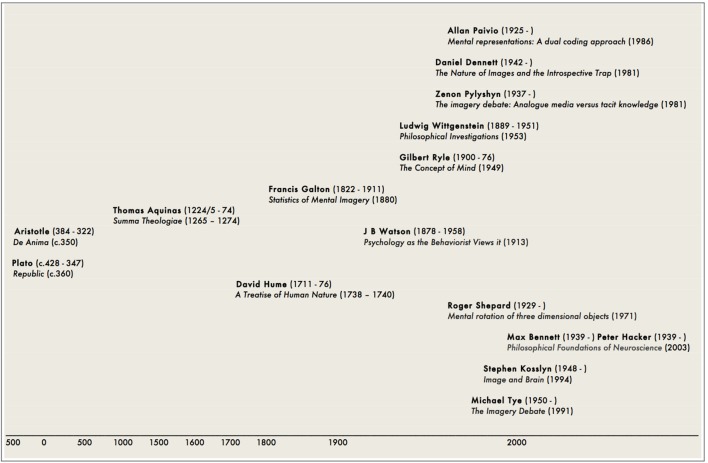
**Indicative chronology of key figures and key works**.

Our aim here, however, is to bridge the gap between this new knowledge and the old by showing that the questions addressed by neuroscientific studies *have a pre-neuroscientific past* that offers to expand the conceptual and contextual horizon of the neuroscientific findings. Accordingly, we will set out how this intellectual history both created the conditions for – and presents challenges to – the scientific endeavor (**Figure 1**).

## Approach

Although many people can experience imagery in all sense modalities (aural, tactile, olfactory, and so on), as well as imagine *without* a sensory component (for example in ‘propositional’ imagining, *that* such-and-such is the case) our focus here is on the most commonly studied sense modality, which is the visual. Even with this limitation in place, the first methodological issue confronting us is one of *scope*: how can we hope to negotiate the two-and-a-half millennia of thought, from theology to philosophy and esthetics, in which some model or theory of the visual imagination has played a role? The prospect is daunting – but the neuroscientific literature itself suggests a method.

Functional imaging studies of the visual imagination give participants mental exercises or tasks to carry out while being scanned. The tasks are various, ranging from imagining specified times on analog clock-faces, then judging the acute angle between the hands (Formisano et al., 2002), to visualizing grids and ‘superimposing’ figures on top (Knauff et al., 2000). But the task that occurs most often is *to imagine a concrete object*, with the instruction delivered either aurally or in writing. In one study, for example, “Two sets of aurally presented nouns ‘concrete’ vs ‘abstract’ were used. … Subjects were instructed to imagine the appearance of the named object during the concrete condition and to listen passively to the words during the abstract condition” (D’Esposito et al., 1997, p. 727). In another, “[s]ubjects were stimulated with a set of 96 … auditory short sentences belonging to … different sensory modalities (Belardinelli et al., 2009, p. 191). The task is also central to the experimental paradigms of Ishai et al. (2000), Handy et al. (2004), Amedi et al. (2005), Gardini et al. (2009), and Daselaar et al. (2010). While imagining can be both willed and unwilled (Pearson and Westbrook, 2015), these tasks, it is important to note, demand a *voluntary* act of imagining.

If the *picturing of a concrete object* is a slim extraction from the imagination’s wide conceptual array, it is exactly its narrowness that makes it a suitable ‘test case’, a means by which to cut into imagination’s intellectual history and extract comparable and contrastive instances. This leads to our other methodological issue, which concerns the criteria on which those instances are selected. Loosely following Dennett’s (1981) description of the “iconophile” viewpoint, the theories and models of visualization that *inform* contemporary scientific analysis are taken to do so because they grant ‘a reality’ to the visual imagination, contending that (1) *images have an important role in cognition*, and (2) *images are either directly or indirectly observable*. We consider these in the first part of the paper. In the second part of the paper we turn to theories of “iconophobes” that pose a challenge to this scientific endeavor – questioning the cognitive importance of imagery, even the existence of mental images, as well as their scientific legitimacy.

Before exploring the first category of imagery theories, we will now sketch a *neuroscientific* account of *picturing a concrete object.* The account will draw on and amalgamate details from several studies that deal with this particular form of imagining, pointing to both the neural mechanisms involved, and to the methods used to explore them. Our emphasis of brain imaging in this account does not imply that it is the only fruitful approach to studying the neurological basis of visual imagery: much can be also be learned, for example, from studies of brain injury (see Farah, 1984).

## Neuro-Imaging Visual Imagination

This account begins with a healthy, right-handed, 33-year old male (Knauff et al., 2000’s median test-participant) lying face-up in the MRI machine, with headphones over his ears. Doing his best not to move, as he has been instructed, he checks with himself that he knows what to do: *listen to each phrase, create a mental image of its content and hold onto that image until you hear the next phrase.* After a last word from an assistant, who exits to a side room, the scanner begins to hum around the participant and the first phrase is piped through his headphones, a strangely genderless voice (as Belardinelli et al., 2009 have it) instructing him to visualize a *candle*. In a moment, another follows, to visualize a chair – but for those few seconds he thinks of a candle (white wax, maybe two fists high, the wick lit). The neural activity that accompanies those thoughts demand oxygenated blood, which is what the scanner detects; neural activity will be inferred from the recordings (this is not to suggest it is a clear path of inference from magnetization to blood oxygen to neural activity: see Buxton, 2009 for an account of the extensive statistical and filtering procedures necessary to overcome the ‘noise’ of the scan and reveal areas of significant activation).

Those inferences will tell us that, when the participant pictures the candle, activity increases in a distributed network of regions across all of his brain. Assemblies of cells in the temporal lobe, involved in attributing and storing information about the appearance of objects (see Fletcher et al., 1995; D’Esposito et al., 1997; Ishai et al., 2000), in turn excite a cascade of neurons in the visual cortices of the occipital lobes. This is roughly a reversal of what happens in perception [as James (1890/1981) was among the first to suggest], where information received in the visual areas of the occipital lobe is projected to visual association areas.

The candle, meanwhile, does not simply ‘appear to’ the participant. He is ‘bringing it to mind’, with all the effort of will that the expression suggests. His task, after all, is to listen to the phrase, *create* a mental image and *hold onto that image.* Generating and maintaining the candle in his mind’s eye depends on activity in the brain’s other two lobes. The active regions form a ‘parieto-frontal’ network that is implicated in ‘top–down’ control – the top–down control in this case consisting in the attention required to form and hold onto the mental image [if attention is indeed a requirement, and not an effect of the task, as Anderson (2011) proposes].

Because external stimulus induces stronger neural activity than visual imagery (see Kreiman et al., 2000), external stimulus tends to ‘win the battle’ for conscious awareness. Another type of top-down modulation may thus be required to bring the candle image to awareness: the suppression of incoming visual information (Jacob et al., 2015). Moreover, without this – which would seem to be achieved by inhibiting the early visual cortex (Kaas et al., 2010) – there is a risk that imagery with an internal source might be mistaken for a percept with an external source, and hallucination would ensue (Frith and Done, 1988); the famous ‘Perky effect’, where external input is misconstrued to be part of mental imagery (Perky, 1910, but see also recent psychophysical replications by, e.g., Bona et al., 2013; Saad and Silvanto, 2013), describes the opposite situation.

This, then, is the knowledge gained (after the writing up, publication, the metanalytic averaging of activation foci, and so on) when the study of imagining takes a technologically sophisticated empirical turn. The neuroimaging results tentatively suggest that visualizing a candle depends on activity across several brain regions: networks based in the parietal and frontal lobes generate and maintain an image which is represented in the temporal lobes, themselves back-projecting information into occipital areas.

With this indicative summary in place, and in pursuit of the conceptual and practical *possibility* of a (neuro-)scientific engagement with visual imagination, we will now jump backward two-and-a-half millennia to the Western intellectual tradition’s primordial account of imagining.

## Foundations of a Scientific Engagement With Visual Imagination

### Classical Natural Philosophy

Plato’s is arguably the first truly philosophical – as opposed to mythological – account of the imagination in the Western tradition. Before Plato, “there was no comprehensive view of the relation of matter to spirit, of the outer to the inner, necessary for an adequate concept of the nature and function of “phantasy”’ (Bundy, 1927, p. 18). In Plato’s system, however, the imagination holds a lowly position: for him, all images are mere copies of things that are themselves copies of divine eternal forms (*Republic* 509 d). Besides – or rather because of – this broad condemnation, Plato makes little distinction between ‘physical’ images, both natural and fabricated, and what would now be called mental images.

Plato (1997, p. 191c,d) does suggest – albeit metaphorically – what we might now call a ‘cognitive’ model of the imagination. In the *Theaetetus* it is suggested that memory might be analogous to a block of wax into which our perceptions and thoughts stamp impressions; in *Philebus*, Socrates declares that there is a “sort of painter in the soul” who “draws in the mind the images of things said”. The painter, it should be noted, merely follows the scribe responsible for the discursive train of thought, the *logos* (Plato, 1997, 39b). For Plato (1997, 511c), the images are incidental ‘epiphenomena’, and moreover, a low kind of thinking: the philosopher uses no ‘object of sense’ but only ‘pure ideas moving on through ideas to ideas and ending with ideas’. For Aristotle, who will not only endorse these models but literalize the metaphors into an “organic” psychological theory (Agamben, 1992, p. 75), reasoning is also the manipulation of ideas; but since for Aristotle, “ideas are not derived from a supersensible realm, either by direct intuition or through the soul’s reminiscences of the ideas it knew in heaven before its incarnation, but by abstraction from the sensible world itself” (Cocking, 1991, p. 18), images become the *essential* intermediary between perception and conception. To understand Aristotle’s notion of what it means to imagine – specifically, to *picture a concrete object* – it is first necessary to appreciate how in his schema one *perceives* an object.

Aristotle’s perceptual theory is dependant on a conceptual framework of ‘hylomorphism’ – literally, “matter-formism”. All things, natural and artifactual, consist of matter and form. Matter is that of which a thing is made, and form is that which makes a thing belong to a particular class of things. On this view wax, wick, and fire are the matter of a candle; the shape – wax vertically arranged, wick running through its center, flame at the top – is that by which the matter is ‘enformed’, and by which we know it to be a candle.

Now, the object-world, for Aristotle, is not static. Things do not sit waiting to be perceived. The forms of objects actually *emanate* from the objects, in ‘movements’ initiated by the objects themselves. An object is sensed when its emanated form impinges on the person’s sense organ and causes the organ to take on the form of the sensed object: when the sense-organ “has been acted upon, it is similar and has the same character as the sensible object” (Aristotle, 1984, 418a3–6). The matter, meanwhile, remains with the object. The analogy (borrowed from Plato) is of a signet ring’s impression in wax: only the shape of the ring is imprinted, not the metal (Aristotle, 1984, 459b25–32). This imprinting is a movement, with *momentum:* the imprint is sent through the blood vessels to the *sensus communis*, the central perceptual organ in the region of the heart, which binds the imprints from the various senses into a coherent spatio-temporal perception.

Veridical perception occurs when an imprint is sent on a clear, uninterrupted trajectory to the central sense organ; “[w]hen one thing has been set in motion,” however, “another thing may be moved by it” (Aristotle, 1984, 428b10–11). The movement of sensation, that is, can give rise to further movements, and this is the movement called *phantasia.* While the *sensus communis* integrates sensory experiences into meaningful perceptions in the presence of the object, the *phantasia* takes that initial movement begun by sensation, and in the absence of the thing perceived produces *phantasmata*: derivatives of sensation, “necessarily similar in character to the sensation itself” (Aristotle, 1984, 428b12), but a side-effect, a knock-on effect, an echo. Picturing a candle, then, would in Aristotle’s account involve movements started by candles themselves. Leaving behind the wax, wick, and flame of their matter, the candles’ sensible forms would have impressed themselves upon the person’s eyes, in turn sending candle-forms along the blood vessels to the heart, before ricocheting off their trajectory to be stored in the heart’s wall (1984, 450a32–b11) in preparation for the *phantasia* to put them in motion once again.

That the motion starts within the person – that the candle is *voluntarily* pictured – is important: it marks off the one picturing *as human*. All animals have *phantasmata*, but deliberative *phantasmata* are confined to those who are able to argue, or those that are “calculative” (Aristotle, 1984, 450a32–b11); the *phantasmata* of the lower animals can only ever be involuntary. However, even voluntary *phantasia*, which is like making or calling up a picture (Aristotle, 1984, 427b 20), is not a creative act: nothing novel or original is generated. As Alan White has pointed out, although *phantasmata* can be called up whenever we wish, Aristotle “lays little stress on its use to envisage possibilities other than the actual” (White, 1990, p. 9). Aristotle’s deliberative imagination is concerned with *planning*, not with counter-factual possibilities.

The kind of picturing just described, we should note in summary, is only one of many roles performed by Aristotelian *phantasmata*: “the soul”, in fact, “never thinks without a *phantasma*” [Aristotle, 1984, 431a 15–20]. They figure in decision-making as much as dreams, hallucinations, and recollections; they are as important to desire and motivation (e.g., Aristotle, 1984, 431a) as they are to communication (Aristotle, 1984, 16a 5–9). Such a universality may, from a modern standpoint, appear unlikely, even alien – but it has a striking contemporary resonance, as we will see in the Conclusion.

### Medieval Philosophy

Where Aristotelianism became the dominant philosophy of Western Christendom, Aristotle’s model of the imagination and views about imagery came to permeate thinking on the subject well into the medieval period. The dominant figure of medieval scholasticism (which held that all knowledge had already been recorded in the writings of church theologians and the Greek and Roman philosophers), Thomas Aquinas developed Aristotle’s account of the psyche in a number of ways. In *Summa Theologiae*, written in the 13th century, he follows Aristotle in considering the imagination to be one of several faculties, but (following the Aristotelian-Arabic tradition of Avicenna and Averroës) divides it into four ‘interior senses’: the *sensus communis*, which unifies the stimuli of external senses, the *imaginativa*, which stores those forms in order to re-compose them, the *memorativa*, which stores those forms as they belong to the past, and the *cogitativa*, which grasps the content of those forms (Aquinas, 1920, p. 78.4). Aquinas also recognized the role of the brain in sensation, where Aristotle believed that it served only to cool the blood (see *Parts of Animals* II.7).

**FIGURE 2:**
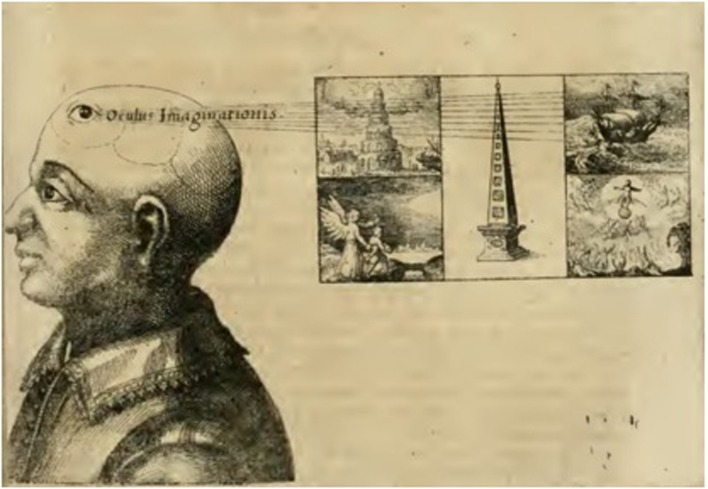
**The *Oculus Imaginationis* (The Eye of the Imagination) as found on the title page of Tractatus One, Section Two, Portion Three of Robert Fludd’s *Ars Memoriae* (Fludd, 1617)**.

Together, these developments urged a model of imagining as a *corporeal* activity. The inner senses, like the outer senses, have bodily organs – and “the power of imagination has its action impeded by damage to the organ [i.e., the brain] … as happens to the phrenetic [literally, an individual with an inflamed brain]” (Aquinas, 1920, 84.7 c23–24). The imagination’s corporeal nature makes it epistemologically crucial, because the intellect has no organ, and so must employ phantasms for understanding to take place. “[E]veryone can experience within oneself,” claims Aquinas, “that when one tries to understand something, one forms certain phantasms for oneself by way of examples, in which one examines, as it were, the thing one is striving to understand. And so it is that when we wish to make someone else understand a thing, we propose examples to him, through which he can form phantasms for himself in order to understand” (Aquinas, 1920, 84.7 c37–43). Indeed, a damaged faculty of imagination impedes the person “from actually understanding even the things he has already acquired knowledge of” (Aquinas, 1920, 84.7 c34–36). Aquinas’s recognition both of the biological basis of mental states (albeit excluding intellectual conception), and the role of imagery in cognition, make his contribution to our history an important one; we will pursue it further in our concluding remarks (**Figure 2**).

### Early Modern Philosophy

Aristotle’s emphasis on the importance of experience came to the fore in the early modern tradition of *empiricism*. As an epistemological position, empiricism’s central tenet is that concepts originate in experience or derive from sensation, rather than being innate. Historically, empiricism appears at many points prior to its more familiar Enlightenment manifestations, and several points after. From an initial reaction against Plato’s rationalism by Stoicism and Epicureanism, an empiricist view of knowledge – i.e., that there is nothing in the intellect that was not previously in the senses – is held by most medieval philosophers. Francis Bacon’s early modern formulation of the principles of scientific induction, where priority is given to observation over deductive reasoning, is a wholly empiricist move.

Although John Locke’s *Essay Concerning Human Under standing* (1690) stands as empiricism’s most elaborate articulation, with its postulation of a ‘blank’ mind at birth progressively filled by experience, David Hume’s systematic development of Locke’s (and George Berkeley’s) positions in his 1739 *A Treatise of Human Nature* is the more relevant to our concerns (Locke, 1975). Hume’s is the most radically empiricist view, holding that there are *only* experiences or ‘perceptions’, to an extent that there is no mind or self that entertains them. The *imagination*, moreover, takes up a role that is novel in its centrality: as the power that links ‘perceptions’ across time it almost comes to occupy the position left empty by the metaphysical self. Before considering what this model of the imagination entails for the act of imagining under investigation, however, we should first sketch out his conception of thought in general.

Hume begins his *Treatise* by calling all mental content, everything of which a person is immediately aware, ‘perceptions’, and goes on to claim that “[a]ll the perceptions of the human mind resolve themselves into two distinct kinds, which I shall call *IMPRESSIONS* and *IDEAS*. Those perceptions which enter with most force and violence we may name impressions … By ideas I mean the faint images of these in thinking and reasoning” (Hume, 2003, 1.1.1.1). When we perceive, then, we are aware of ‘impressions’, and when we think, we are aware of ‘ideas’. The central claim here, however, with ramifications throughout the *Treatise*, is that ideas are “faint images” of impressions. This means that things imagined or conceived derive only from things sensed, and are “nothing but copies and representations” (i.e., “re-presentations”) of them (the contrast with Plato, for whom all images, whether mental or physical, are copies of things that are themselves copies of divine eternal forms, is stark). The ideas, moreover, are “faint”: indeed we find that they are distinguishable from impressions only by the ‘vivacity’, or by the ‘force and liveliness’, with which they ‘strike the mind’. “That idea of red,” for example, “which we form in the dark and that impression which strikes our eye in sunshine differ only in degree, not in nature” (Hume, 2003, 1.1.1.5). The idea of a candle, similarly, is just a dimmer, weaker, “faint[er] image” of the impression of a candle; there is no *intrinsic* difference between a candle imagined and a candle perceived.

Two faculties serve to re-present impressions to ourselves as ideas: memory and imagination. Although remembered and imagined ideas also differ in relative vivacity (the idea of a past event “flows in upon the mind in a forcible manner”, an imagined event appears “faint and languid”), remembered and imagined ideas differ most fundamentally by their *composition*. Memory’s ideas are direct reproductions of impressions: “When I shut my eyes and think of my study, the ideas I form are exact representations of the impressions I felt when I was in my study; every detail in one is to be found in the other” (Hume, 2003, 1.1.1.3). The imagination, however – and here the departure from Aristotelian doctrine should be marked – “isn’t bound to keep the same order and form as the original impressions had”, and may produce ‘complex’ ideas by joining parts of several impressions together. The imagination is at “liberty [to] transpose and change its ideas”, as literary descriptions of mythical creatures suggest (Hume, 2003, 1.1.4.5): I have never (and could never have) seen a Pegasus, but I have seen horses, and I have seen wings, and imagination combines them.

Although the imagination can “separate ideas and then re-unite them in whatever form it pleases”, it does not do so randomly. There are some “universal principles” – more psychological regularities – that cause “bonds of union” to form among ideas. Simple ideas, which are direct copies of impressions, associate into complex ideas by *resembling* one another, by being *contiguous* in time or space, or by standing in relation of *cause and effect*. It is these principles of imaginative association that allow our test subject to picture a complex idea, as they respond to the instruction *to see a candle*.

To answer the question of how our participant is able to do that, we must first answer the question of how they acquire the Idea of a candle in the first place, and the answer is through custom, or habit. Encountering groups of impressions enough times – e.g., wax, wick, flame – leads the different instances to be associated with one another. A name becomes associated with that group of impressions via the principle of *contiguity*: the word ‘candle’ is heard when the participant sees a short, white, cylindrical, wax thing, when they see a tea light, and when looking at a long, tapering, red thing (Broughton, 2000). Thus, “[w]hen we have found a resemblance among several objects, that often occur to us, we apply the same name to all of them, whatever differences we may observe in the degrees of their quantity and quality, and whatever other differences may appear among them” (Hume, 2003, 1.1.7.7) After the participant has acquired a custom of *wax-wick-flame*, the hearing of the name ‘candle’ “revives the idea of one of these objects, and makes the imagination conceive it with all its particular circumstances and proportions”: a candle in general, but appearing to the participant as one of those candles whose visual impression was contiguous in time with one of those resembling auditory impressions (exactly how such a thought can be functionally and semantically of a *general* candle, while appearing to the thinker as a *particular* candle, it must be noted, is never explained by Hume).

Meanwhile, that the idea of a candle is subjectively differentiated from the impression of a candle according to their relative vivacity – by a difference of *degree* – has a serious consequence for our imagining participant. Because idea and impression are the same *kind* of thing – i.e., perception – and perceptions “succeed each other, and never all exist at the same time” (Hume, 2003, 1.4.6.2), the imagined candle is not something that interrupts the visual field or ‘superimposes itself*’* over awareness of an exterior world. Being imagined grants the candle no special status: the candle is merely one in a stream of perceptions, some dimmer than others, that “successively make their appearance; pass, re-pass, glide away, and mingle in an infinite variety of postures and situations. …. They are the successive perceptions only, that constitute the mind” (Hume, 2003, 1.4.6.4). Further – and here the implications of such a radical empiricism as Hume’s are most manifest – the perceptions are not entertained *by* any subject: there is no consistent ‘self’ that perceives an imaginary candle, having perceived the instruction to imagine one beforehand. It is the associative imagination that autonomously impels the train of perceptions – *and it is the perceptions that constitute the mind*.

### Early Scientific Psychology

When applied to mental phenomena such as acts of imagining, the experimental process – of making observations, gathering data, and drawing implications for a prior hypothesis – would seem to be obstructed at the first stage: other people’s images are simply not available to observation, after all. Thus scientific research into mental imagery begins with standardized instructions to imagine, introspect and provide *verbal* accounts of one’s imagery. Sir Francis Galton’s questionnaire *on visualizing and other allied activities* – issued in November 1879, results published in *Mind* in 1880 – was the first of these.

Galton’s primary interest was not visualizing itself, however. Rather, the test examined the capacity to recollect a visual scene as an index of the “peculiarities of the mental visions of different persons”: its ultimate aim was to “elicit facts that shall define the natural varieties of mental disposition in the two sexes and in different races, and afford trustworthy data as to the relative frequency with which different faculties are inherited in different degrees” (Galton, 1880, p. 302). The questionnaire was one of a number of means and methods Galton devised for recording mental as well as physical characteristics (sensory acuity, for example, as much as bodily proportions and fingerprints) that would contribute to an understanding of the determining effects of heredity. Wider investments aside, the questionnaire provided a solution to the problem of how to register, measure, and statistically analyze individual differences in mental phenomena that was highly innovative – although by no means unproblematic (as Schwitzgebel, 2011, for one recent example, argues). It was distributed first to friends and relatives, then to some schoolboys, then to 100 of Galton’s male acquaintances, of whom a majority were “distinguished in science or in other fields of intellectual work” (Galton, 1880, p. 304). The questionnaire begins by asking the subjects to “think of some definite object – suppose it is your breakfast-table as you sat down to it this morning – and consider carefully the picture that rises before your mind’s eye.” They are then asked, “1. *Illumination* – Is the image dim or fairly clear? Is its brightness comparable to that of the actual scene? … 2. *Definition* – Are all the objects pretty well defined at the same time, or is the place of sharpest definition at any one moment more contracted than it is in the real scene?” (Galton, 1880, p. 302) Consideration proceeds to “Color”, the “Extent of the field of view”, “Distance of images”, and “Command over images”; further questions enquire about instances of imagery in other modalities.

The results, Galton found, displayed a wide variation. They ranged from one respondent who claimed “I can see my breakfast table or any equally familiar thing with my mind’s eye, quite as well in all particulars as I can do if the reality is before me”, to another who admitted, “My powers are zero. To my consciousness there is almost no association of memory with objective visual impressions. I recollect the breakfast table, but do not see it” (Galton, 1880, p. 310).

Galton drew two main conclusions. One was that “scientific men” as a class have feeble powers of visualization; this was because “an over-readiness to perceive clear mental pictures” is “antagonistic to the acquirement of habits of highly generalized and abstract thought”. While Galton is an advocate of visualizing – it should be “judiciously cultivated by as yet undiscovered educational techniques” – the prejudices of the age seem to be at play. Imagers and non-imagers are divided by gender, class, and age: it is “women and intelligent children” who have vivid imagery and are happy to describe it, while it is “men who think hard” (the scientists) who do not think in images. These claims have not been confirmed by later research, 20th- and 21st-century studies (Isaac and Marks, 1994; Brewer and Schommer-Aikins, 2006) having failed to replicate his results.

Galton’s other main conclusion was that the study itself was a success: it had “proved [the] facility of obtaining statistical insight into the processes of other persons’ minds”. Indeed, “[t]he conformity of replies from so many different sources… and the evident effort made to give accurate answers, have convinced me that it is a much easier matter than I had anticipated to obtain trustworthy replies to psychological questions” Galton’s (1883/1907, p. 60) confidence in introspection was born out as experimental psychology developed in the latter part of the 19th century – in the work of Wilhelm Wundt, Gustav Fechner, and Hermann von Helmholtz – into a distinct academic discipline based on the use of quantitative introspective methods. Whether the replies to Galton’s questions were as “trustworthy” as he thought, however, is another matter – which we will discuss in Section “Challenges to a Scientific Engagement with Visual Imagination” – and one that ultimately contributed to the demise of introspectivist psychology.

### Cognitive Science

Where, in the early 20th-century, introspectivist psychology had fallen to behaviorism as the dominant approach, behaviorism’s reign in turn came to an end in the 1960s, as cognitive psychology developed models of underlying mental structures supported by objective evidence. The behaviorist account was left wanting by the demonstration of aspects of thought that functionally *depended* on mental phenomena, and imagery in particular.

Alan Paivio’s (1963) work on memory was key in this regard. His research showed that it was easier to remember concrete nouns, such as ‘truck’ or ‘tree’, that *can* be imaged, than it is to remember abstract nouns, such as ‘truth’ or ‘justice’, that are difficult to image. Corroborated by self-reports provided by research participants, the role of imagery as the effective mediator of associative learning, and as an explanatory construct, was established. Where Paivio’s work used *memory* tasks to suggest the cognitive processes that their completion required, Shepard and Metzler (1971) used ‘mental chronometry’: the measuring of the amount of time taken to carry out various cognitive tasks. Their ‘mental rotation’ experiment showed that the time taken to determine whether two rotated geometric figures were the same or different corresponded linearly to the angle they needed to be rotated to make the comparison. They interpreted the result as showing that participants were mentally rotating one of the views to see whether it matched the other.

If so, it seemed that mental imagery *in itself* was fundamental to thought, that the ‘pictorial’ qualities of imagery could no longer be regarded as epiphenomenal, in Stephen Kosslyn et al.’s (2006, p. 6) striking simile, “like the heat thrown off by a lightbulb when one reads (which plays no role in the reading process)”. Indeed it was Stephen Kosslyn who became the main advocate of this ‘analog’ model, where internal representations share the same format as external stimuli, being irreducibly *images* of what they represent. Kosslyn himself had been lead to this position by the results of ‘mental scanning’ experiments, which demonstrated that the time it took to shift attention from one part of an imaged object to another corresponded to the distance between those two points on the physical object itself (Kosslyn et al., 1978). This was taken to show that imagery shares the same mechanisms as perception and that the internal representations involved in imagining represent by being *depictive*, inasmuch as they are spatially structured.

This was not, however, the only possible explanation of these results. Pylyshyn (1981) accounted for them by appealing to ‘tacit knowledge’: subjects in the experiments would have unconsciously tried to emulate what they *thought* they would have done in the corresponding perceptual situation, e.g., taking more time to scan greater distances across the imagined object (an explanation itself rejected on empirical grounds by Jolicoeur and Kosslyn, 1985). Pylyshyn’s contrary interpretation made up a part of a vociferous and long-running disagreement over the nature of mental imagery. Around the same time that Paivio, Kosslyn and others were developing their approaches, advances in computing began to suggest computational models and conceptions of human information processing. It is from the perspective of computer science that Pylyshyn (1973, p. 1) refuted explanations of experimental findings that relied on imagery, and rejected outright the “picture metaphor” of visual mental imagery. Instead, claimed Pylyshyn, mental imagery should be understood to consist in “abstract mental structures to which we do not have conscious access and which are essentially conceptual and propositional, rather than pictorial, in nature. Such representations are more accurately referred to as symbolic descriptions than as images in the usual sense” (ibid.). More on this ‘descriptionalist’ position below; for the moment it is enough to say that the so-called ‘imagery debate’ ran until advances in neuroimaging techniques in the late 20th-century provided converging evidence that, for many (see Pearson and Kosslyn, 2015), confirmed the depictive model of imagery. Using PET, Kosslyn et al. (1993) showed that when subjects perform visual imagery tasks the occipital visual cortex is activated, analogously to how it activates when objects are physically present. Key to Kosslyn’s argument was the presentation of evidence (e.g., Slotnick et al., 2005) that visual imagery activates ‘retinotopic’ visual cortices that preserve the spatial layout of the retina. In perception, this means that the pattern of light stimulating the retina will be repeated there as activation; in visual mental imagery, it means that there will be a correspondence between the spatial form of the mental image and the spatial pattern of neural activity. This is evidence, according to Kosslyn, that “imagery relies on representations that depict information, not describe it” – conclusive evidence, in other words, “that mental imagery relies on actual images” (Kosslyn et al., 2001, p. 639). Although Pylyshyn (2003) in the early 2000s continued to reject Kosslyn’s interpretation – arguing that mental images and topographical patterns of activation in the early visual cortex do not convincingly correspond more recent findings, that, for example, rely on machine learning algorithms using patterns of activation in the early visual cortex to reconstruct what is being visualized (Naselaris et al., 2015), would seem to make the ‘analog’ model of mental imagery very difficult to refute.

What was at stake for cognitive science in the visualizing of a candle, then, were fundamentally opposed theories of the mind’s operation. Is the candle in the test-subject’s ‘mind’s eye’ there as an epiphenomenal side-effect of his mind’s essentially computer-like operation, or is it an instantiation of the very medium through which he thinks? The findings of cognitive science and the analyses of philosophy have interlaced over such questions. We will now turn to the philosophers’ interventions.

### Analytic Philosophy

What has recent philosophy made of our test-subject picturing a candle? By way of an answer, we will look firstly at philosophical responses to the debate in cognitive science that we have just encountered, over the representational format of imagery. We will then discuss a related attempt to sharpen the *concept* of imagery itself – specifically, asking if imagery is necessarily ‘experiential’ – before suggesting how the two lines of enquiry align with fundamentally opposed positions on the nature of mental imagery.

Following Michael Tye, we can distinguish three philosophical views emerging in response to the kind of theory about mental images that Aristotle and the British Empiricists, and latterly Stephen Kosslyn, put forth (i.e., that they are essentially picture-like, and represent by being so). One is the behaviorist view, which we will discuss in more detail in Section “Challenges to a Scientific Engagement with Visual Imagination”, below; another is ‘Adverbial Theory’, proposed by Tye himself. From comparative semantic analyses of phrases about mental images, Adverbial Theory concludes that, ultimately, there are none. Grammatical similarity between statements such as “the subject has an image of a candle” and “the subject has a candle” would suggest a logical similarity – that the subject *can* have an image of a candle, just as she can have *a* candle. But the similarity breaks down into serious problems. Can two persons have one and the same image (like they can a candle)? If they cannot, and mental images are not the kind of things than can be ‘had’, are they then nonphysical? And if mental images are nonphysical objects, then “how did they emerge in the evolution of matter?” (Dennett, 1991, p. 29). Such conundrums suggest to Tye that philosophers have been misled by the grammar of ordinary language, assuming grammatical form indicates logical form, to the false conception that there are images to be ‘had’.

The third view is ‘descriptionalism’. Zenon Pylyshyn is the principal exponent in cognitive science; philosophers advancing a descriptionalist account of imagery include J. M. Shorter and Daniel Dennett. The central claim is that mental images represent objects in the same way that linguistic descriptions represent objects. As Tye points out, this does not mean that during imagery spoken language is in some way present in the imager’s mind; rather, the claim is about the way the phenomenon of mental imagery gets encoded in the brain, that mental images’ “neural code … is, in some important respects, language-like” (Dennett, 1991, p. 27). So mental images are not a *special* form of picture-like, depictive thought. Indeed, Sterelny (1986) suggests philosophical descriptionalism may be partly motivated by a desire for a unified model of the mind: if other propositional attitudes like memory, belief, and desire are best understood as being structurally sentence-like, a linguistic account of mental images forms a more coherent approach. Moreover, according to its advocates, descriptionalism explains characteristics of mental imagery that are a challenge to accounts of mental images that figure them as inner pictures. *Indeterminacy*, for example. As both Shorter (1952) and Dennett (1969) point out, descriptions often leave things unspecified: I may say that there is a candle in front of me, for example, without specifying whether or not the wick is lit. When mental images are “noncommittal” in this way – my visualization of a candle is still *of a* candle without it specifying whether the wick is lit or not – it would seem that representation via description can account for the indeterminacy. Elsewhere, Dennett (1991, p. 55) observes that “[d]ifferent listener’s phenomenology in response to the same utterance can vary almost ad infinitum without any apparent variation in comprehension or uptake”. Considering the variation in mental imagery that might be provoked in two people who hear the sentence ‘Yesterday my uncle fired his lawyer’, Dennett finds that comprehension or ‘uptake’, as could be confirmed by subsequent paraphrasing and responses to questioning, remains determinate; the mental image is contingent, and thus *epiphenomenal* as far as understanding is concerned (Plato’s painter in the soul merely illustrating the scribe’s discursive train of thought is the distant antecedent here).

A related question that recent philosophy asks of our putative image of a candle is whether or not it is ‘experiential’. For Christopher Peacocke all sensory imagining (i.e., involving imagery) is experiential, in that when one imagines a candle, one is imagining the experience of perceiving a candle, not a candle on its own. As Peacocke (1985, pp. 22–23) puts it, “If S sensorily imagines an F … then S sensorily imagines from the inside perceptually experiencing an F … in the imaginary world. Martin (2003, p. 403) calls this the *Dependency Thesis*, and, following Peacocke, argues that sensory imagining – “those distinctive episodes of imagining or imaging which correspond to our use of the distinct senses” – is *necessarily* imagining sensing. By representing a candle, Martin claims, mental imagery inherits the phenomenal properties of experiencing a candle (Martin, 2003, p. 406). The *dependency* (of imagination on perception) explains the way that visualizing is perspectival: that I seem to only be able to picture a candle from a certain point in space in relation to the candle is due to perceptions being from certain points in space, and imaginings being imagined perceptions.

Peacocke and Martin’s thesis is directly challenged by Paul Noordhof, who accounts for the fact that mental images always represent their objects from a certain perspective by appealing to what he calls the *Similar Content Hypothesis*. This holds that perceptions have contents, which are the features of an object conveyed to the subject by perceiving it – i.e., the candle’s white wax, its height of around 20 cm, its wick aflame – and that these will be similar to equivalent modes of sensory imagining. An imagining of a candle combines the content with a possible point of view drawn from our knowledge of egocentric space, a “*supposition*’ (Noordhof, 2002, p. 439) that the imagined candle is presented relative to a single viewpoint. The candle, concludes Noordhof, is visualized from a certain imaginary point not because the subject is in some way aping perceiving a candle but because the visualization is informed by extra-visual knowledge of how things look in relation to oneself; thus imagining a candle does not need or ‘depend’ on perception.

We can see that Noordhof’s view on whether sensory imagining is imagined sensing has essential correlations with the descriptionalist view of mental images: both hold that visual mental imagery is underwritten by information that is not itself in a visual format, whether it be Pylyshyn’s ‘tacit knowledge’, a language-like neural code, or a combination of ‘supposition’ and ‘content’ (which itself amounts to a kind of ‘appearance report’). Conversely, Peacocke and Martin’s *dependency thesis* about sensory imagining entails, like the ‘depictive’ account of mental imagery’s representational format, a direct relationship with perception. According to Martin, one’s mental image of a certain object *is of* that object because it inherits the phenomenal properties of *perceiving* such an object; integral to Kosslyn’s account of mental imagery is the neuroscientific finding that visualizing utilizes – *depends on* – visual perception’s physiological apparatus. And, to continue the knowledge-perception opposition, we might counter Dennett’s descriptionalist explanation of imagery’s representational indeterminacy with the simple observation that mental images can be vague, *just like percepts can*. We will see in the next section which side of the knowledge-perception opposition most challenges the scientific investigation of imagery.

## Challenges to a Scientific Engagement With Visual Imagination

### Behaviorist Psychology

The primary conceptual and methodological challenge to scientific work on imagining comes from the *behaviorist* movement in psychology and philosophy that flourished in the first half of the 20th-century – but also from associated, ‘behavioristic’ attitudes and conceptions that have continued into the present.

Behaviorism, at least in its historical form, rejects outright imagining and mental images as objects of scientific study. “Psychology as the behaviorist views it”, ran Watson’s (1913) manifesto article, “is a purely objective experimental branch of natural science.” Psychology deluded itself in thinking it had made mental states the object of observation. As far as Watson was concerned, psychology should no longer be conducted in terms of “consciousness, mental states, mind, content, introspectively verifiable imagery, and the like”; instead, it should be done in terms of “stimulus and response, in terms of habit formation, habit integrations and the like” (Watson, 1913, p. 160). The manifesto dedicates a lengthy footnote to the problem of mental imagery, or as Watson calls it, “centrally aroused visual sensations”, where it is hypothesized that all of the “so-called ‘higher thought’ processes” (including imagery) are in fact “faint reinstatements of the original muscular act”, occurring as “motor habits in the larynx”. As 1928’s *The Ways of Behaviorism* reiterates, when a person “closes his eyes” and says “I see the house where I was born, the trundle bed in my mother’s room where I used to sleep – I can even see my mother as she comes to tuck me in”, that person is “merely dramatizing”: the behaviorist “finds no proof of imagery in all this. *We have put all these things in words, long, long ago”* (Watson, 1928, pp. 76–77). On Watson’s (1928) view, then, not only is imagery not a proper object for scientific enquiry, but ‘first-person’ reports of the experience of it are invalid too. According to Watson, when we think we are imagining or picturing a certain scene we are in fact having “a conversation about [it] either to ourselves (thought)” – (potentially) observable as tiny “subvocal” movements of the throat – “or with some one else (talk)” (ibid.). Later versions of behaviorism – specifically Skinner’s (1953) – were more lenient regarding imagery: it *could* be granted existence, but only as a form of deeply hidden, effectively unobservable, covert behavior. If Skinner’s constitutes a *psychological* behaviorism – that attempts to explain all behavior in terms of stimuli, responses, and so on – and Watson’s constitutes a *methodological* behaviorism – a normative theory about the conduct of psychology, where private mental states do not form proper objects of empirical study – there remains an *analytical* or *logical* behaviorism, exhibited by the mid-20th-century philosophy of Gilbert Ryle and the later Ludwig Wittgenstein.

### Analytical Behaviorism

Ryle and Wittgenstein’s behaviorism is really an effect of the application of their particular ‘ordinary language’ approach to philosophy of mind. The central contention of ordinary language philosophy is that language is the means by which “thinking goes public” (Brann, 1991, p. 156). Consequently, philosophical analysis consists of examining not immediate experience (as is the case in, for example, the phenomenological tradition) but the ways in which language is used, and specifically the ways in which mental phenomena are commonly spoken about. Ryle’s (1949) *The Concept of Mind* takes common expressions of consciousness experience – like ‘seeing with the mind’s eye’ – to indicate an erroneous belief in what amounts to a “ghost in the machine”. Wittgenstein’s (1953) *Philosophical Investigations* is more circumspect: there is not the active repudiation of mental events or processes, but rather a disinclination to credit them with explanatory power. Consequently Wittgenstein is not concerned with the *experience* of imagining: “[w]e are not analyzing a phenomenon (e.g., thought) but a concept (e.g., thinking) and therefore the use of a word” (Wittgenstein, 1953, p. 383). When analyzing a concept such as imagination it does not help to “point” to any particular experience of it – this would make us expect “a wrong kind of answer” (Wittgenstein, 1953, p. 370). But as much as Wittgenstein moves the object of analysis from phenomenon to concept, he also wants to minimize the import of the phenomenon itself, not least in the case of comprehension: “It is no more essential to the understanding of a proposition that one should imagine anything in connection with it, than that one should make a sketch from it” (Wittgenstein, 1953, p. 396). The point seems to be that being a mental process grants imagining or imaging *no special status –* it could be substituted with a sketch or indeed a verbal description of what is imaged. Hence there is first-person authority in respect of mental images – we can only know what is being imagined by what the imaginer reports – because the ‘interior’, mental aspect is elided into the report. “Suppose”, writes Wittgenstein, “that someone were to draw while he had an image, or instead of having it, though it were only with his finger in the air. He could be asked “Whom does that represent?” And his answer would be decisive. It is quite as if he had given a verbal description: and such a description can also simply take the place of the image” (Wittgenstein, 1953, 177e). Logically, the image on its own, whether drawn or imagined, is ambiguous – it takes a description to identify it, which in turn makes the image itself redundant (note the connection with Paul Noordhof’s supplementary “supposition”, the non-visual knowledge that the visualization relies on). On a psychological level, the consequence is not just that attention is focused on the expression of what one imagines, but that the mental image is taken to *coincide with* that expression, rather than be a private entity which is then described: the description can take the place of the image.

If Wittgenstein’s account constitutes an attack on the imagination, or mental imagery at least, Gilbert Ryle’s account is “demolitionist” (Warnock, 1976, p. 153). The locus of Ryle’s assault is Chapter VIII of *The Concept of Mind* (1949), where it is the notion of mental images – discreet copies of things once seen – as existing somehow ‘in’ the mind, which is rejected. As Ryle puts it: “the familiar truth that people are constantly seeing things in their minds’ eyes and hearing things in their heads is no proof that there exist things which they see and hear, or that the people are seeing or hearing” (Ryle, 1949, p. 222). Just as a murder performed on stage in a theater has no victim and is not a murder, “so seeing things in one’s mind’s eye does not involve either the existence of things seen or the occurrence of acts of seeing them” (Ryle, 1949, p. 223). Ryle’s claim can be best understood as a rejection of both Cartesian dualism, with its necessary implication that mental processes or events are played out in a ‘private theater’, and – especially in this case – Hume’s empiricist epistemology. Because seeing in the mind’s eye is not *seeing* at all, it makes no sense to speak of it being on a continuum of “vivacity” with visual perception; thus, “[t]o say that an action is a mock-murder is to say, not that a certain sort of mild or faint murder has been committed, but that no sort of murder has been committed” (Ryle, 1949, p. 228). Ultimately – again undermining the ‘seeing things’ model of imagining, as Ryle calls it – to imagine *x* is merely “one among many ways of utilizing [my] knowledge” of *x.* Seeing an object in my mind’s eye is one of the things which my knowledge of that object enables me to do: “describing it in words is another and a rarer ability; recognizing it at sight in the flesh is the commonest of all” (Ryle, 1949, p. 242).

### Wittgensteinian Critique

The methods and tenets of ‘ordinary language’ philosophy have been applied recently to cognitive neuroscience. Bennett and Hacker’s (2003) *Philosophical Foundations of Neuroscience* sets about a conceptual critique of cognitive neuroscience’s methods and claims. Like Ryle, their object of attack is Cartesian dualism, but in this case it is the dualism of neuroscience when it commits the ‘mereological fallacy’: ascribing to parts of humans properties that make sense to predicate only of whole humans, i.e., “the brain sees” and “the left hemisphere thinks”. “Human beings, but not their brains, can be said to be thoughtful or thoughtless” (Bennett and Hacker, 2003, p. 73). The problem with cognitive neuroscientists construing brains and brain hemispheres as believing, perceiving, imagining, and so forth, is that such constructions prevent an understanding of human behavior any better than “construing clock cogs as giving the time allows us to understand clock behavior” (Burgos and Donahoe, 2006, p. 75) – and ultimately lead to meaningless research presuppositions, justifications, and interpretations.

**FIGURE 3:**
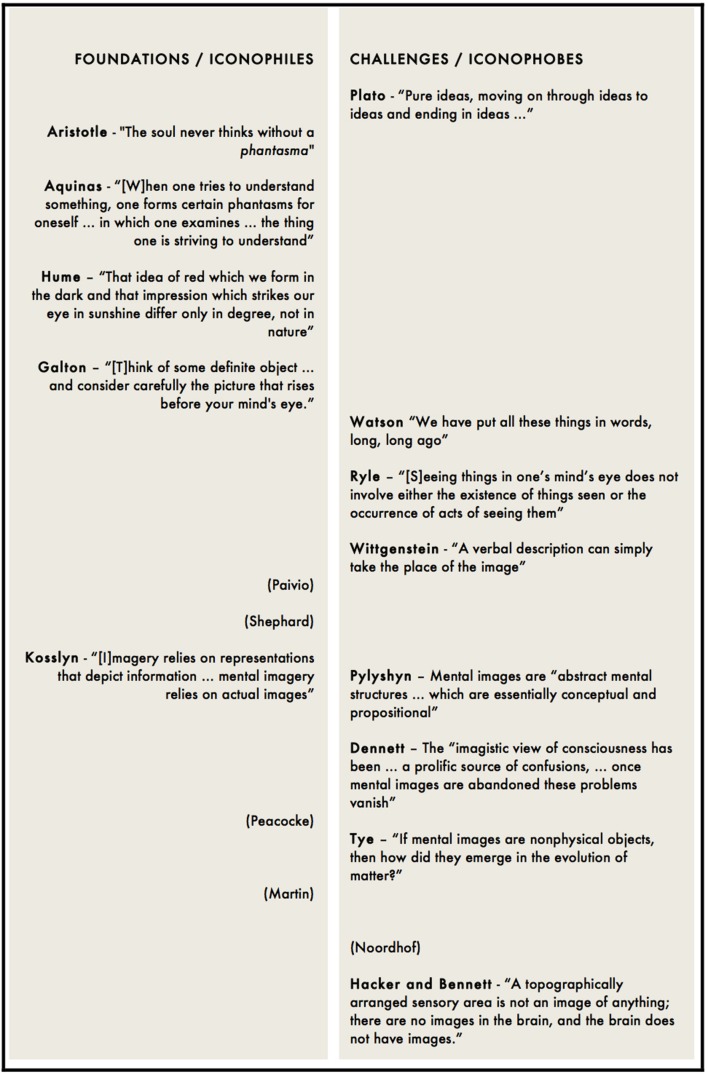
**Table of “iconophile” positions, that provide the foundations for neuroscientific engagement with mental imagery, and “iconophobic” positions, that challenge it**.

Hacker and Bennett’s analysis of cognitive neuroscience’s claims includes those made about notions of the *imagination*: its relation with creativity, perception, belief, and the place of ‘mental imagery’ in its conceptualization. The points made there are largely recognizable from Ryle and Wittgenstein: mental images are a major source of conceptual confusion because, contrary to common parlance, we do not in fact “have” them (because they are not things that can be possessed); they need not be involved in imagining; and, reiterating their refutation of the mereological fallacy, the *brain* does not ‘have’ imagination. The same logic undermines foundational psychological treatments of mental imagery. Francis Galton’s (1880) questionnaire *on visualizing and other allied faculties* is predicated, say Hacker and Bennett, on the false belief that mental images are objects ‘in’ the mind, available for inspection. Shepard and Metzler’s (1971) mental rotation task, similarly, does not stand as ‘evidence’ for mental imagery. For Hacker and Bennett, the problem is not only that such evidence for mental imagery is indirect – mental images are not being measured or detected in any way – but that *indirect* evidence is no evidence at all. Neuroscientific claims to identify the neural concomitants of mental states are similarly dismissed: such concomitants are ‘merely’ inductive evidence. The only criterion for whether someone has a visual image of something, Bennett and Hacker conclude, reiterating Wittgenstein’s claim of first-person authority in respect of mental images, “is that he says that he has and can say how he visualizes what he imagines” (Bennett and Hacker, 2003, p. 180) (**Figure 3**).

## Foundations, Challenges, Insights, and Reflections

### Foundations

The study of visual imagery in contemporary cognitive neuroscience belongs to a long tradition in natural philosophy; indeed, the neuroscientific work can even be seen as a technologically sophisticated empirical turn in that lineage. The intellectual history that creates the conditions for the scientific endeavor does so in two main ways. Firstly, its *attitude* to visual imagination grants it an observable ‘reality’ and a central function in cognition. Aristotle’s understanding of *phantasia*, the capacity that enables us to visualize things in their absence; Hume’s account of ‘ideas’ as faint copies of impressions; Galton’s early measurements of the vividness of imagery; Paivio’s exploration of the relationship between imageability and memorability; Shepard’s demonstration that manipulations of imagery mirror manipulations of the items they represent; Kosslyn’s depictive theory of imagery all, in their *conceptual affirmation* of visual mental imagery, prepare the ground for the *empirical* study of its neural correlates.

Secondly, these historical moments – particularly the earlier ones – inform and anticipate a distinctly *scientific* approach to visual mental imagery. Aristotle’s conviction that ultimate reality lay in the physical world, knowable through *experience*, is the primordial example. It makes possible a science or *epistêmê* that begins in sense perception and builds up to an understanding of the necessary and invariant features of the world. At the same time, Aristotle admits a fundamental uncertainty about how to study the soul. Does it belong to ‘natural science’ (i.e., physics) – since various psychological states, including anger, pity, love, and hate, all involve the body in obvious ways? (Aristotle, 1984, 403a16–28) – or, considering that the ‘higher’ processes of mind or *nous* do not seem to involve the body to the same extent, is a natural scientist inadequate to the study of the soul (Aristotle, 1984, 1026a4–6)? The uncertainty echoes through the history (and pre-history) of psychology.

There is an irony in imagination’s being given such a pivotal role in David Hume’s epistemology: the *Treatise* is to no small extent motivated by a desire to *correct* the imagination’s undue influence on philosophy. It is the imagination, claims Hume, that begets ‘unscientific’ metaphysical systems and the ‘fancy’ that suggests hypotheses about worlds beyond that of experience; the ancient philosophers are disparaged for being seduced by “every trivial propensity of the imagination” (Hume, 2003, 1.4.3.11). As the introduction makes clear, the *Treatise* is directly intended to constrain metaphysics in order to make way for a “science of man” – namely, an investigation into human psychology on a solely empirical basis – and rein in the imagination accordingly.

Francis Galton’s technique for making mental phenomena available to measurement and statistical analysis could be said to make good on Hume’s promise. It proved to be influential on many levels, not least by its several (more readily quantifiable) progeny, including Betts’s (1909) ‘Questionnaire upon Mental Imagery’, Gordon’s (1949) ‘Test of Visual Imagery Control’, and Marks’s (1973) Vividness of Visual Imagery Questionnaire (VVIQ). And while behavioral techniques such as mental rotation have done more to confirm the ‘reality’ of imagery, Galton’s questionnaire stands as the first attempt to overcome the methodological problems caused by imagery’s inherently private nature. In confirming the visualized candle, for example, as an entity whose values of “*Illumination … Definition* … [and] *Coloring”* reveal themselves on scrutiny, it affirms that such acts of imagination *are amenable* to scientific analysis: the visualized candle *can* be observed, the observations *can* be articulated and treated as data to be systematically gathered, implications *can* be extracted.

### Challenges

As we have seen, some historical thinkers have questioned the role of imagery in cognition and cast doubt on the feasibility of studying this salient but elusive aspect of our experience. Behaviorism, in its psychological, methodological and analytical forms, holds that experience *per se* is not an appropriate target for science. Hacker and Bennett, more specifically, argue that work seeking the neural basis of psychological function in the functioning of the brain commits a logical fallacy – hunting for an explanation in small parts of the body for functions that properly belong to the whole. Pylylshyn and the philosophical ‘descriptionalists’ have questioned whether ‘thinking in pictures’ has any special status, arguing that every kind of thought is fundamentally abstract or propositional. How destructive are these ‘iconophobic’ challenges to contemporary imagery science?

While the measurement of behavior is at the heart of scientific psychology, the renaissance of interest in consciousness over the past several decades reflects the failure of proponents of behaviorism to persuade the intellectual community that talk of ‘experience’ is no more than a redescription of behavior. John Searle’s terse remark that such theories ‘have left out the mind’ strikes many readers as apposite. The fundamental nature of ‘experience’ remains mysterious and controversial, but the successful exploration of its neural correlates reinforces the everyday intuition that our ‘inner lives’ are rich and real, and undermines philosophical arguments (like Wittgenstein’s, Ryle’s, or Tye’s) that dispute the reality of our inner lives on the basis of the language by which they are commonly spoken about. It is highly probable that visual imagery depends on brain regions which are also used in visual perception, pointing to a distinctively depictive mode of mental representation. This is not to deny the possibility of important commonalties, at neural and computational levels, between all forms of representation in the brain (as current computational models indeed hold – for example Eliasmith’s (2013), where the mechanisms that underlie multiple cognitive functions are sought).

Meanwhile the ‘mereological fallacy’ does not appear to be a major barrier to continuing research: *people*, not brains, it is true, see, remember and imagine, but some parts of people, and not other parts, are crucial to these functions. Refining our understanding of the biological basis of experience, by probing these relationships, commits no logical fallacy. It seems to us that, following the commentator’s analogy, one should indeed examine the cogs of a clock in order to understand how it gives the time.

Bennett and Hacker’s claim, that the only criterion for whether someone has a visual image “is that he says that he has”, would seem to be rendered untenable by such powerful demonstrations of imagery’s biological basis as the ability to use fMRI-measured activation patterns to reconstruct what a person is visualizing (Naselaris et al., 2015). Ultimately, the ‘iconophobic’ positions we have described – besides appearing, often as not, as attempts to explain post-hoc empirical results produced by ‘iconophilic’ researchers – seem unable to withstand neuroscientific engagement.

### Insights

The philosophical history we have reviewed points to the richness and complexity of the concept – and the activity – of imagining. Following the lead of many recent researchers, we have focussed in this paper on the task of visualization, the formation of an image of an object in its absence. This relatively simple task is massively intricate in neural terms, and we still have much to learn about its neural basis. But ‘imagination’ can engage other senses than vision, and other modes of brain function than sensation, as in motor imagery; occur in propositional as well as sensory forms; involve creativity; allow the contemplation of possible worlds. Scientists hoping to plumb the depths of imagination will need to take account of this richness and complexity which may be most fully revealed in a literature that neuroscientists – understandably – seldom consult.

### Reflections on the ‘Prehistory’ in the Light of Imagery Science

Throughout much of the history of thought, ‘philosophy’ included areas of knowledge that we currently assign to science. Reviewing the thoughts of Aristotle, Aquinas, or Hume on the imagination, it seems likely that they would have been fascinated and delighted by contemporary research in neuroscience. They were early psychologists: their models were concerned as much with empirical as with conceptual aspects of the mind. To some extent, therefore, the contemporary neuroscience of imagery is the natural heir of a philosophical tradition; indeed, the findings of imagery science, and the theories built around them, make several moments in its prehistory strikingly prescient.

That imagery shares processing mechanisms with like-modality perception, and that visual mental imagery has been conceptualized as a weak or ‘noisy’ form of top–down perception (Pearson et al., 2015), echoes Hume’s conviction that there is no *intrinsic* difference between something imagined and something perceived, and that ‘ideas’ are distinguishable from ‘impressions’ by the lesser ‘vivacity’ or ‘force and liveliness’ with which they ‘strike the mind’. Aquinas’s (1920, 84.7 c34–36) observation that “the power of imagination” is “impeded by damage to [the brain]”, which impedes in turn the person from “understanding even the things he has already acquired knowledge of” would seem to anticipate such cases as reported by Brain (1954) and Zeman et al. (2010), where stroke or head injury precedes sudden loss of the ability to summon visual imagery, even of familiar routes or faces. And contemporary theories of ‘embodied’ cognition, which propose that all forms of cognition involve modality-specific mental simulations, and imply that imagery plays a functional role in all cognitive events, give the Aristotelian tenet that “the soul never thinks without a *phantasma*” [Aristotle, 1984, 431a] a fresh pertinence.

Such connections play out in support of the actuality and relevance of mental imagery. As we have seen, however, there have always been ‘imagery skeptics’, thinkers who have doubted that imagery plays any real or important or measurable role in our mental lives. The stark opposition between the views of imagery enthusiasts and imagery denigrators raises the question of whether these two groups of thinkers may have been working, in some sense, with different data. Reisberg et al. (2003) have shown that the views of participants in the ‘imagery debate’ were influenced by the vividness of their own imagery: those with vivid imagery were more sympathetic to the view the imagery is picture-like, and more likely to judge imagery worthy of further research. We wonder if, as Berman (2008) suggests, something similar may apply to the differing perspectives of philosophers of imagery whose views may also have been influenced by their varying, personal experiences of imagery. The centrality of imagination in David Hume’s epistemology, alongside his report that when he shut his eyes and thought of his study, the “ideas” he formed were “exact representations of the impressions [he] felt when [he] was in [his] study” (Reisberg et al., 2003, 1.1.1.3), are a case in point.

If, as Tye (1991) argues, pre-scientific thinkers have been over-reliant on their subjective experience, the great benefit of science is that it can confront subjective impression with objective fact. To a striking degree, contemporary cognitive neuroscience is finding objective, neural correlates for both robust and more elusive aspects of our experience – for example, our experience of illusion (Zeki et al., 1993), of hallucination (Ffytche et al., 1998), even of shivers down the spine (Blood and Zatorre, 2001). Unlike Aristotle, Hume or even Watson and Wittgenstein, we are in a position to triangulate experience, behavior and biology, linking the experience of imagery to measurable aspects of behavior and processes in the brain. We hope that this paper will help to make this triangulation a worthy continuation of the long and fascinating history of thought about the ubiquitous experience of imagery.

## Author Contributions

MM conducted the research and, with AZ, wrote the manuscript; all authors contributed to the analyses and revision of the article at several stages. All authors approved the final version of the manuscript.

## Conflict of Interest Statement

The authors declare that the research was conducted in the absence of any commercial or financial relationships that could be construed as a potential conflict of interest.
